# How to improve influenza vaccine coverage of healthcare personnel

**DOI:** 10.1186/s13584-016-0122-3

**Published:** 2016-12-16

**Authors:** David J. Weber, Walter Orenstein, William A. Rutala

**Affiliations:** 1 Department of Hospital Epidemiology, University of North Carolina Health Care, 2163 Bioinformatics, CB #7030, Chapel Hill, NC 27599-7030 USA; 2 Division of Infectious Diseases, UNC School of Medicine, Chapel Hill, NC USA; 3 Division of Infectious Diseases, Department of Medicine, Emory University School of Medicine, Atlanta, GA USA

**Keywords:** Occupational health, Healthcare personnel, Vaccines, Influenza

## Abstract

Influenza causes substantial morbidity and mortality worldwide each year. Healthcare-associated influenza is a frequent event. Health care personnel (HCP) may be the source for infecting patients and may propagate nosocomial outbreaks. All HCP should receive a dose of influenza vaccine each year to protect themselves and others. This commentary will discuss the study recently published in the IJHPR by Nutman and Yoeli which assessed the beliefs and attitudes of HCP in an Israel hospital regarding influenza and the influenza vaccine. Unfortunately, as noted by Nutman and Yoeli in this issue many HCP in Israel choose not to receive influenza immunization and many harbor misconceptions regarding their risk for influenza as well as the benefits of influenza vaccine. We also discuss proven methods to increase acceptance by HCP for receiving an annual influenza vaccine.

## Background

Protecting healthcare personnel (HCP) and patients from acquiring an infectious disease via person-to-person spread requires strict adherence to key infection prevention and occupational health recommendations [1]. First, hand hygiene before and after every patient contact and at other appropriate times [2]. Second, use of Standard Precautions by HCP when providing care for all patients (i.e., use of appropriate personal protective equipment to prevent contact with infectious body fluids or aerosols) [3]. Third, rapid evaluation of patients with a known or suspected communicable disease and institution of appropriate isolation precautions (i.e., Contact, Droplet and/or Airborne) [3]. Finally, immunizations of all HCP to prevent vaccine preventable diseases [1, 4–8]. In developed countries this would include: mumps-measles-rubella, varicella, tetanus-diphtheria-acellular pertussis, hepatitis B (HCP who have potential exposure to contaminated blood or body fluids), *Neisseria meningitidis* (microbiologists), and influenza. Additional vaccines (e.g., polio, hepatitis A, *Bacillus* Calmette-Guerin) may be recommended in specific countries and for relief workers [6–8].

Influenza occurs globally with an annual attack rate estimated at 5% to 10% in adults and 20% to 30% in children [9]. Infection may result in hospitalization and death, especially among high-risk groups (e.g., the very young, older adults, chronically ill, and/or immunocompromised). The World Health Organization (WHO) estimates that these annual epidemics result in 3 to 5 million cases of severe illness, and about 250,000 to 500,000 deaths [9]. It is important to remember several aspects of influenza epidemiology: influenza is easily spread from person-to-person; it can affect anybody in any age group; seasonal influenza is a serious public health problem that causes severe illness and death in high risk populations; prior influenza disease does not assure immunity because the virus frequently mutates from year-to-year (antigenic drift; although antiviral drugs are available for treatment, influenza viruses can develop resistance to drugs; and, influenza vaccination is the most effective way to prevent influenza [9].

Healthcare-associated influenza is a major worldwide problem for several reasons [10–12]. First, hospitals provide care to persons at high-risk for morbidity and mortality if they acquire influenza including healthcare-associated infection including neonates, older persons, those with chronic Illnesses (e.g., diabetes, heart disease, asthma, lung disease) and immunocompromised persons. Second, nosocomial outbreaks are frequent and their control remains challenging. Third, the diagnosis of influenza is commonly missed because many infected and infectious persons are asymptomatic or mildly symptomatic and the clinical signs and symptoms of influenza can be confused with similar illnesses caused by a variety of other pathogens. Further, persons with influenza may be infectious prior to the onset of symptoms. Fourth, molecular analyses have revealed transmission between patients and HCP, and HCP and patients. HCP have served both as sources of nosocomial outbreaks and propagators of healthcare-associated outbreaks. Fifth, immunization of HCP (4 cluster randomized trials and 4 observational studies) conducted in long-term care or hospital settings has demonstrated that immunization of HCP demonstrates a “significant protective association for influenza-like illness and laboratory-confirmed influenza’ [13].

As noted by the Society for Healthcare Epidemiology of America (SHEA), influenza vaccination of HCP serves several purposes: (1) to prevent transmission to patients, including those with a lower likelihood of vaccination response themselves; (2) to reduce the risk that the HCP will become infected with influenza; (3) to create “herd immunity” that protects both HCP and patients who are unable to receive vaccine or unlikely to respond with a sufficient antibody response; (4) to maintain a critical societal workforce during disease outbreaks; and (5) to set an example concerning the importance of vaccination for every person [11].

For the reasons listed above, influenza immunization annually of all HCP is recommended by the World Health Organization [6], the Centers for Disease Control and Prevention [4, 5, 10], almost all countries in Europe [7, 8], other countries around the world [8], and many professional organizations. Further, the Israel Minister of Health includes coverage rates among its ongoing quality measures for healthcare organizations, and this may be helping increase coverage rates. Nevertheless, influenza immunization of HCP is challenging for two key reasons: (1) the vaccine efficacy varies year-to-year depending on how well the strains in the vaccine match with the circulating strains and (2) the vaccine must be provided each year. Two key issues regarding achieving high influenza vaccine coverage of HCP are discussed below. First, what has been reported in the scientific literature regarding why HCP are willing or not willing to receive influenza vaccine? Second, what methods to improve influenza coverage among HCP have been reported in the peer-reviewed literature?

### Assessing why HCP are or are not willing to receive influenza vaccine

In a recent article published in the Israel Journal of Health Policy Research, Nutman and Yoeli reported on an assessment of the knowledge, perceptions and attitudes concerning influenza vaccination among HCP in a large tertiary care academic hospital in Israel [14]. The authors obtained their information by means of an anonymous survey completed by 468 HCP representing all categories of staff. The overall frequency of influenza immunization among these HCP was 42% (physicians, 56%; nurses, 41%; allied health professionals, 37%; and, administrators and support personnel, 30%). Key findings were as follows: (1) most HCP understood that influenza is widespread and can have severe complications including death; (2) only ~82% agreed that hospital personnel are at an increased risk of contracting influenza because of their job; (3) only ~62% believed that influenza immunization is the only effective way to prevent infection; (4) only about 60% disagreed that vaccine side effects can be more severe than influenza; (5) ~50% believed that influenza vaccine can cause influenza; (6) >10% believed that pregnant women should not be immunized; (7) only ~30% thought that if they were not immunized they would become ill with influenza; (8) less than 50% recommend influenza vaccine to their patients; and (9) ~50% were in favor of mandatory immunization of HCP. In the multivariate analysis, items that were independent predictors of immunization were beliefs that: vaccine effectively prevents influenza, HCP are at increased risk of influenza, contracting influenza is likely in the absence of immunization, and HCP might transmit influenza to their families.

The study by Nutman and Yoeli provided similar information to studies in other developed countries. The influenza vaccine vaccination rate of 42% in their hospital is similar to rates reported from many European countries [8], but well below the 77.3% reported in the US [15]. For both Israel and the US, physicians and nurses reported a higher frequency of influenza immunization than allied health professionals and nonclinical HCP. Other surveys which assessed the knowledge, perceptions and attitudes concerning influenza vaccine by HCP have reported similar results as the study by Nutman and Yoeli [16–19]. Beliefs encouraging influenza immunization have generally included a desire to protect oneself and to a lesser extent to protect patients. Other factors favoring immunization include free and convenient immunization, being previously immunized, and peer-pressure and/or support by senior administration. Beliefs reducing the likelihood of immunization have included fear of adverse events, misconception that influenza vaccine can cause influenza, belief that the person is not at risk, doubt that influenza is a serious disease, concern that the vaccine is ineffective, fear of injections, and that times/locations for immunization were inconvenient. It is highly disturbing that HCP frequently hold misconceptions regarding influenza such as the lack of severity of influenza and risks for generally healthy HCP, as well as holding misconceptions regarding the vaccine such as the vaccine can cause influenza and that adverse events are common. Space limitations preclude a detailed review of the science debunking these misconceptions. However, by way of example, we can note that in Israel, influenza vaccine has been shown to reduce influenza in HCP [20]. Further, multiple studies which have evaluated the frequency of adverse events following influenza immunization of HCP have reported that they are mild and transient [21–23]. The vast majority of influenza vaccines used around the world are inactivated, many which are split or subviron which cannot possibly cause influenza.

### Improving influenza coverage among HCP

As already noted the public health authorities in many countries, as well as many professional organizations, recommend that all HCP should receive a dose of influenza vaccine each year. For example, the Israel Ministry of Public Health includes influenza vaccine coverage among its ongoing quality measures for healthcare organizations which will help increase coverage rates. This public health recommendation is based on sound public health and the peer-reviewed literature. Given this well-founded recommendation, what methods have been demonstrated to improve influenza vaccine coverage among HCP? The 2005, SHEA Guideline listed the following barriers and solutions to HCP influenza immunization [24]: (1) Inconvenient access to vaccine (solution: off-hours clinics, use of mobile vaccination carts, vaccination at staff and department meetings, and provision of adequate staff and resources; (2) Cost (solution: provision of free vaccine); (3) concerns for vaccine adverse events (solution: targeted education including specific information to dispel vaccine myths); and (4) other (solution: strong and viable administrative leadership, visible vaccination of key leaders, active declination of HCP who do not wish or cannot be vaccinated, accurate tracking of individual HCP and unit-based compliance of HCP with vaccination, and surveillance for healthcare-associated influenza). Since this publication, substantial additional research has been published on the effectiveness of different methods to improve influenza vaccine acceptance by HCP (Table 1) [24–35].

**Table 1:** Interventions That Improve Influenza Vaccine Coverage for Healthcare Personnel

• Mobile carts
• Free vaccine
• Adequate staff resources for vaccine campaign
• Education on benefits and risks (or lack of risks) for immunization
• Incentives for immunization
• Immunizations available on nights and weekends
• Immunization available at convenient locations (e.g., meetings, common areas)
• Administrative support including visible vaccination of key personnel
• Tracing vaccination by individual healthcare providers and hospital units with regular feedback to healthcare providers and administrators
• Sanctions for failure to be immunized
• Employment conditional upon receipt of vaccine

Several methods for increasing HCP compliance with influenza immunization deserve further discussion; the use of declination forms, the requirement that HCP who are unable or unwilling to receive influenza vaccine wear a surgical mask while providing patient care or while on a patient unit, and “mandatory” influenza immunization. The 2005 SHEA Guideline on influenza vaccination of HCP recommended as one modality to increase influenza uptake, the use of a declination form to be signed by HCP who were unwilling to accept vaccine [24]. This declination form described the risk to the HCP and their patients from the HCP refusing immunization. Subsequent research has demonstrated that use of such forms was associated with only a modest increase in vaccine use by HCP, even when combined with other strategies to increase vaccine coverage. Multiple studies demonstrated that the introduction of declination forms continued to lead to vaccine coverage <80% [36–38] and often <70% [39]. The 2010 revised SHEA Guideline on influenza vaccination of HCP included the statement “the use of statements (i.e., declination forms) should not be viewed as the primary method for increasing vaccination rates’ [11].

Another intervention that has been recommended is to require unvaccinated HCP to wear a surgical mask during the influenza season [11]. Several potential issues related to the masking requirement have been raised. First, implementation of such a policy is logistically challenging (i.e., developing methods to identify those HCP required to wear a mask during clinical care). It has proved difficult to develop a simple method to identify such HCP without stigmatizing those HCP who chose not to be vaccinated or were unable to be vaccinated due to vaccine contra-indications. Second, few studies that have assessed the success of this policy have reported on the number of non-compliers and what penalties were assessed for non-compliance.

Research has revealed that hospitals which include disincentives for HCP refusing influenza vaccine have higher rates of influenza vaccine coverage [39–41]. Examples of disincentives have included requirement to sign a vaccine declination form and a requirement that non-immunized persons wear a surgical mask while on clinical units. The most successful strategy to improve HCP influenza vaccine coverage has been to make receipt of vaccine as a condition of employment (i.e., “mandatory” immunizations). Hospitals employing this strategy exempt HCP with a contra-indication to immunization and some also exempt HCP with a religious objection. Multiple reports of hospitals that use this strategy have reported vaccine coverage rates >95% [15, 39, 42, 43]. Increasing numbers of hospitals in the US now require receipt of influenza vaccine as a condition of employment (HCP with a contra-indication are usually exempt) [15]. Concern has been raised about the ethics of “mandatory” immunization of HCP. However, multiple professional societies have endorsed that employment as a HCP should be conditional on willingness to receive influenza vaccine, as vaccine protects both the HCP and the patient.

## Conclusions

Influenza causes substantial morbidity and mortality worldwide each year. Healthcare-associated influenza is a frequent event. HCP may be the source for infecting patients and may propagate nosocomial outbreaks. All HCP should receive a dose of influenza vaccine each year to protect themselves and others. Unfortunately, as noted by Nutman and Yoeli in this issue many HCP in Israel choose not to receive influenza immunization and many harbor misconceptions regarding their risk for influenza as well as the benefits of influenza vaccine. Multiple proven methods can be used by healthcare facilities to improve influenza vaccine coverage among HCP (Table 1). The only proven method to reliably achieve a coverage level >95% is to require influenza immunization as a condition of employment.

## Abbreviations

HCP: Healthcare personnel
SHEA: Society for Healthcare Epidemiology of America
WHO: World Health Organization
